# Use of anesthetics associated to vasoconstrictors for dentistry in patients with cardiopathies. Review of the literature published in the last decade

**DOI:** 10.4317/jced.50590

**Published:** 2012-04-01

**Authors:** María A. Serrera Figallo, Rocío T. Velázquez Cayón, Daniel Torres Lagares, Jose R. Corcuera Flores, Guillermo Machuca Portillo

**Affiliations:** 1 Department of Dentistry. University of Seville; 2 ……..; 3 ….

## Abstract

Objective: The use of local anesthetics associated to vasoconstrictor agents in dentistry is thoroughly justified and is widely extended, but we cannot ignore the fact that anesthetic infiltration poses risk of complications throughout the dental treatment period. The objective of the present review is to document the reported effects the use of the local anesthetics most widely employed in dentistry, with or without association to vasoconstrictor agents may have in patients with any sort of cardiopathy. Study Design: We have searched for randomized clinical trials on the assessment of the cardiovascular effects of local anesthetics used in dentistry, without limits as regards age or sex, conducted in patients with any type of cardiopathy which were published during the last decade and were index-linked in Cochrane, Embase and Medline. Results: We have found six randomized clinical trials index-linked in Medline and Cochrane in the past ten years. These trials compare different types of anesthetics: lidocaine 2%, mepivacaine 2%, prilocaine 2% , associated or not to different vasoconstrictor concentrations such as adrenaline or felypressin. The cardiopathies affecting the patients included in the different trials range from hypertension, ischemic heart disease, arrythmias, chronic coronary disease to heart transplantation. Conclusions: The use of anesthetics associated to vasoconstrictor agents is justified in the case of patients with cardiopathies (once we get over the period in which any type of dental manipulation is contraindicated) and in controlled hypertensive patients. In any case, we must be very careful with the choice and execution of the anesthetic technique, being it possible to use a dose between 1.8 and 3.6 ml, on a general basis. Further studies are necessary to establish the effects of these drugs on severe hypertensive patients or in patients with other more advanced cardiopathies.

** Key words:**Vasoconstrictor agents, epinephrine/adverse effects, local anesthetics, dental restoration, oral surgery, cardiovascular diseases, coronary arteriosclerosis, heart disease, hypertension, arrhythmias, coronariopathy.

## Introduction

The use of anesthetics associated to vasoconstrictor agents is justified in the clinical practice of dentistry: it delays the absorption of local anesthetics and increases safety as lower doses of anesthetic are required (which diminishes its potential toxic risk). The use of Catecholamine type vasoconstrictors in certain patients is a controversial issue, especially in the case of patients with cardiopathies and/or diabetes and more precisely in those with coronariopathies and severe hypertension (1).

In order to avoid these risks associated with the use of catecholamines, researchers have focused their efforts in searching for vasoconstrictor agents with a different chemical structure. Nowadays, only felypressin is marketed in Spain (2-L-Phenylalanine-8-L-lysine-vasopressin). It could be an adequate alternative to catecholamine type vasoconstrictors in the following circumstances: cardiopathies and especially ischemic cardiopathies, patients with high plasma concentrations of endogenous catecholamines (feochromocytoma) or thyroid hormones (primary or secondary hyperthyroidism), patients under treatment with substances susceptible of causing dangerous interactions with catecholamines (beta blocking drugs, tricyclic antidepressants, MAOIs antidepressants, sympathomimetic drugs such as cocaine, etc) or allergy to vasoconstrictor agents themselves (2-4).

One of the most delicate and risky actions throughout any type of dental treatment is anesthetic infiltration. If we consider as well the great number of pharmacologic effects infiltrated substances have (anesthetics themselves and the adrenergic vasoconstrictors used to increase biodisponibility and to reduce anesthetic toxicity), it is obvious that the specialist confronts a situation he must know and control thoroughly. This situation becomes even more delicate when the patient suffers from any cardiopathy.

Within the category of patients with cardiovascular pathologies we include.

- Hypertensive heart disease, which in turn comprises two varieties, namely, hypertensive urgency and hypertensive emergency. The latter may pose a vital risk for the patient and it requires urgent parenteral hypotension treatment (5). Stage A hypertension is observed in patients with levels > 180/110mmHg without organic damage or associated factors. Stage B hypertensive patients show some systemic associated risk factor (tobaccoism, dyslipidemia, over 60 yrs or a previous history of vascular processes) with the exception of diabetes mellitus and no signs of cardiovascular affectation. Stage C patients suffer from cardiovascular diseases, organic damage and / or diabetes mellitus but do not show any other risk factors.

- Ischemic heart disease: first of all, we must bear in mind the fact that patients who have suffered acute myocardial infarction have a high risk of suffering a new infarction, and very often severe arrhythmias. Also, the risk of a second infarction during non cardiac surgery is known to reach 27% during the first months after infarction, 11% between months 3 and 6 and it drops to 5% from month 6 onwards. As a result, dental treatment is not indicated up to 6 months after the episode of acute myocardial infarction (6,7).

- Arrhythmias: arrhythmia is defined as any disorder of your heart rate or rhythm. As regards heart rate we may find two opposing situations, bradyarrhythmia (heart rate less than 60 beats per minute) and tachyarrhythmia (heart rate more than 100 beats per minute). In these patients preoperative evaluation must be detailed in order to assess the origin of the arrhythmia, the need of prior stabilization of the patient before the treatment, the risk of arrhythmia during treatment and the possibility of avoiding the use of local anesthetics associated to vasoconstrictor agents (8).

- Patients who have undergone heart transplantation: survival rate of these patients has increased remarkably with the introduction of immunosuppressive drugs such as cyclosporine. The side effects of such drugs include a high incidence of gingival hyperplasia. We must determine the correct dose of local anesthetic to be administered to this type of patients (9).

In line with different authors, we can state that in the case of healthy subjects the number of anesthetic carpules that can be administered is extrictly limited (15). Therefore, the greatest evidence so far reported on the effect of local anesthetic associated to vasoconstrictor agents used in dentistry on the cardiovascular system has been observed in healthy subjects (16).

Nevertheless, the number of patients with cardiopathies who visit our clinics in increasing and the use of anesthetics associated to vasoconstrictor agents in this type of patients poses an interesting dichotomy. On the one hand, they imply greater safety and on the other hand their use may provoke cardiovascular complications.

The aim of the present study is to search for and conduct a critical evaluation of recent reports on the effect and safety for the cardiovascular system of local anesthetics associated with vasoconstrictor agents used for dentistry in patients with cardiopathies.

## Material and Methods

We have searched for studies published in the last decade (January 2000 through December 2010) which have been index-linked in Cochrane, Embase and Medline, in the latter using the Pubmed search tool.

We established the following search paremeters: randomized controlled clinical trials conducted in humans without limit as regards age of male/female sex with any type of cardiopathy or cardiovascular disease written in English and Spanish and published in the last decade. The search words used were: vasoconstrictor agents, epinephrine/adverse effects, local anesthetics, dental restoration, oral surgery, cardiovascular diseases, coronary arteriosclerosis, heart disease, hypertension, arrhythmias, coronariopathy, using the Boolean operator OR to combine search words.

Finally, the results obtained were manually filtered reading the abstracts published in the above mentioned sources in order to subsequently obtain the original version of the selected articles.

## Results

We have found six randomized clinical trials: Ezmek and colls. (2010), Elad and colls. (2008), Fernán-dez-Cáceres and colls. (2008), Neves and colls. (2007), Conrado and colls. (2007), Meechan and colls. (2002) (9-14). These reports focus on different types of anesthetics: lidocaine 2%, mepivacaine 2%, prilocaine 2% with or without association to different concentrations of vasoconstrictor agents such as adrenaline or felypressin.

The cardiopathies studied in the different trials range from hypertension to ischemic heart disease (stable angina pectoris, myocardial infarction of more than 6 months of evolution, coronary artery bypass of more than 3 months of evolution, congestive heart failure) including arrhythmias, chronic coronary disease and heart transplantation.

 Table 1 shows the most significant information of the above mentioned six reports: study design (prospective randomized controlled), inclusion and exclusion criteria (differentiating diseases and their treatment), total number of patients (between 30 and 65), local anesthetics employed with or without association to vasoconstrictor agents and number of patients in each group, anesthetic dose (between 1 and 4 carpules of 1.8ml), parameters considered (ECG, BP and HR, in most cases), intervals and differences observed, if any.

**Table 1:**
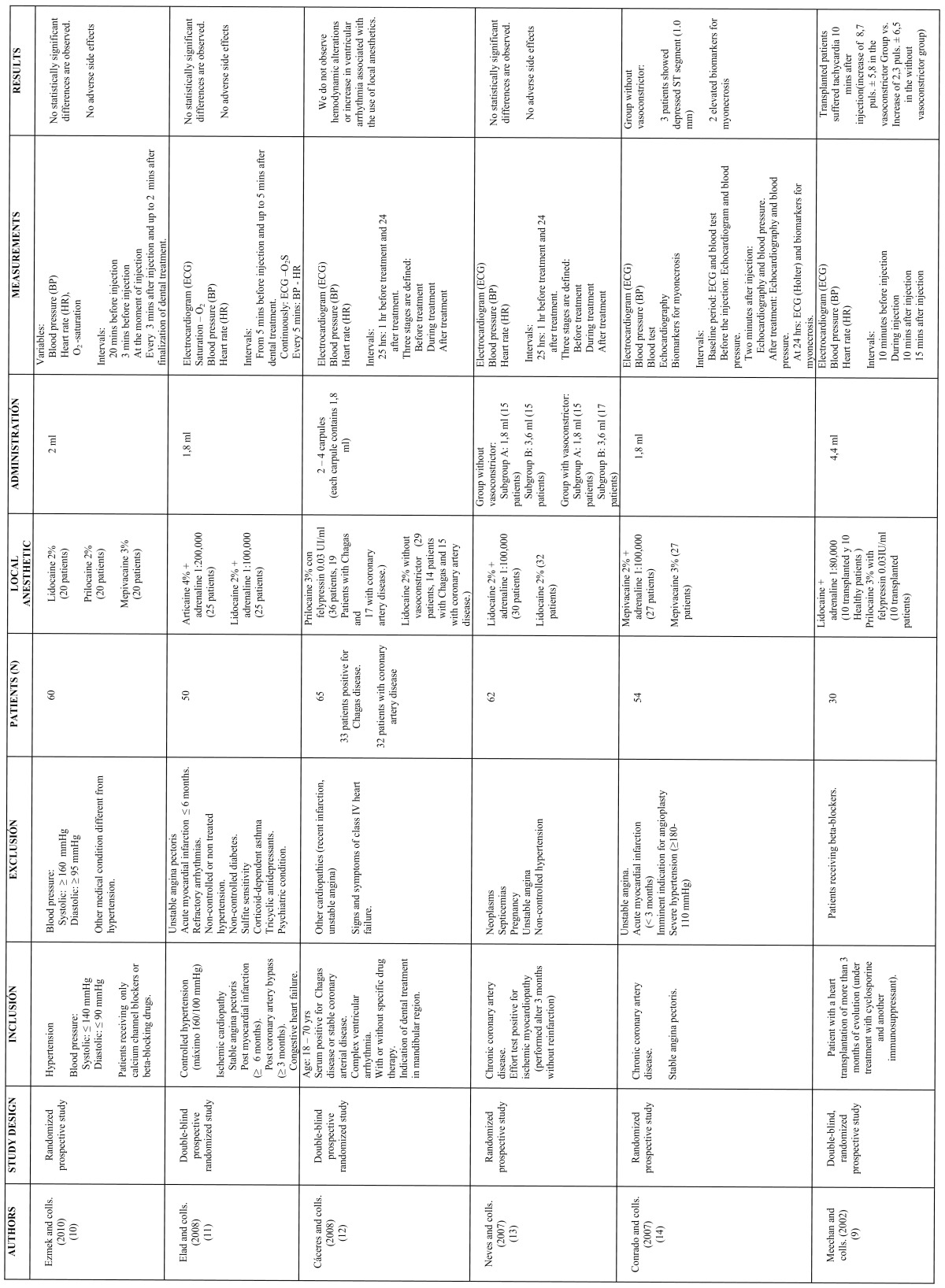
Results of search about clinical randomized trials in use of anesthetics associated to vasoconstrictors for dentistry in patients with cardiopathies.

## Discussion

Epinephrine or adrenaline is one of the most widely used vasoconstrictor agents in association with local anesthetics in dentistry. This substance provokes the constriction of the blood vessels in the mucosa and skin, which favours latency period and reduces the dissemination of the local anesthetic due to the action of alpha-adrenergic receptors. But the β1-adrenergic receptors of epinephrine will provoke an increase in heart rate and the β2-adrenergic receptors will cause vasodilation in muscles and internal organs. However, only a few reports exist which relate the use of non-selective beta-adrenergic blocking agents and severe hypertensive side effects in dentistry (17).

A good number of publications assess the effects of different anesthetics on certain cardiovascular parameters. Most of these reports have been carried out in healthy subjects and in general the alterations observed in parameters such as arterial tension (mainly), heart rate and others are not significant when comparing the use of anesthetics with and without vasoconstrictor agents. In these studies we observe a slight increase in the value of the above mentioned parameters which the authors usually attribute to the stress of the patients and to the rise in the levels of endogenous epinephrine or adrenaline (18).

The last systemic review of this subject, carried out by Bader and colls. in 2002 dealing with the cardiovascular effects of the use of epinephrine in dentistry in hypertensive patients showed that neither systolic nor diastolic blood pressure increased, even in the case of non-controlled patients (19).

Since then, 6 other studies have been published which contribute level 1 evidence (randomized clinical trials) in this field of study. These studies have expanded their field of research to include patients with ischemic cardiopathies (stable angina pectoris, acute myocardial infarction, patients with bypass) as well as patients with heart transplantation or chronic coronary artery disease. As a result of these recent studies we are nowadays aware of the effect local anesthetics with or without association to vasoconstrictor agents have on a wider range of pathologies related to the cardiovascular system.

The first conclusion we must mention is that in most of these studies we did not observe significant differences between patients with cardiopathies treated with anesthetics with or without association to vasoconstrictor agents, and when such differences were statistically detected, the authors did not consider them critical from a clinical point of view (as is the case of the study by Meechan and colls. or Conrado and colls. (9,14).

The anesthetics included in our study are widely used in dentistry (prilocaine, lidocaine, mepivacaine, articaine) in association or not with vasoconstrictor agents such as adrenaline or felypressin. This indicates that no new drugs have been introduced which could increase safety in their use with patients with cardiopathies. The doses under study are similar to those administered to healthy subjects, so we must assume that in these studies local anesthetics are administered under normal conditions.

Although some studies report the use of invasive techniques to control hemodynamic and cardiovascular variables, the variables measured in the studies we have selected were arterial tension, heart rate, oxygen saturation and the use of electrocardiogram (20). Only one of the studies searches for a possible cardiac damage not shown by means of these variables using other techniques: biomarkers of myonecros, echocardiography or blood test (14).

Generally, authors start to control these variables before the onset of treatment and finish once it has ended establishing three well differentiated periods (11): before treatment (baseline period) which in the reports under study is set from 5-10 mins to 1 hr before treatment; during dental treatment; after dental treatment which may last from a few minutes after treatment up to 24 hrs with a Holter. 50% of the studies analysed use this technique to carry out postoperative control of patients (12-14). The use of the Holter as well as a longer follow-up period after injection of the anesthetic is thoroughly justified by the complexity of these patients and the risk of accident, even of a subclinical type (which we must detect) in the hours following the administration of the anesthesia.

The exclusion criteria ban from the studies those patients with temporary conditions which contraindicate the use of the treatment (acute myocardial infarction of less than three or six months of evolution (11,14) or those with severe varieties of the pathologies under study (severe hypertension, class IV heart failure (10,12). So far, there is no information available on the effects local anesthetics associated to vasoconstrictor agents may have on the cardiovascular system of these patients, therefore we must avoid their use, or be extremely cautious in case of administration. In our opinion, such information is necessary and should be obtained by means of studies where patients, due to the complexity of their lesions and the risk of severe complications, are increasingly controlled and even hospitalized during the whole study period.

Likewise, we cannot ignore the fact that the greater the complexity of the patients the wider the range of pathologies we will encounter and the greater the variety of drugs they will be administered. This in turn, can also provoke cardiovascular alterations in case of administration of local anesthetics associated to vasoconstrictor agents (9).

From the review of all the above mentioned studies we can conclude that in patients with cardiopathies (once we get over the period in which any type of dental manipulation is contraindicated) and in controlled hypertensive patients, the use of local anesthetics associated to vasoconstrictor agents is justified. In any case, we must be very careful with the choice and execution of the anesthetic technique, being it possible to use a dose between 1.8 and 3.6 ml, on a general basis. Further studies are necessary to establish the effects of these drugs on severe hypertensive patients or in patients with other more advanced cardiopathies.
